# Symmetric Dimeric Structure and Ligand Recognition of CutR, a LysR-Type Transcriptional Regulator from *Mycobacterium* sp. Strain JC1

**DOI:** 10.3390/ijms262110533

**Published:** 2025-10-29

**Authors:** Hyo Je Cho, Ka Young Lee, Hyun-Shik Lee, Beom Sik Kang

**Affiliations:** 1 Department of Biochemistry, Chungbuk National University, Cheongju 28644, Republic of Korea; hyojec@cbnu.ac.kr; 2 School of Life Science and Biotechnology, Kyungpook National University, Daegu 41566, Republic of Korea; ky03175@naver.com (K.Y.L.); leeh@knu.ac.kr (H.-S.L.)

**Keywords:** LysR-type transcriptional regulator, protein–ligand interaction, dimerization, crystal structure, CO dehydrogenase, *Mycobacterium*

## Abstract

Mycobacteria possess carbon monoxide dehydrogenase (CO-DH) to utilize CO as an energy source and to resist host defense mechanisms. The expression of the CO-DH gene is regulated by CutR, a LysR-type transcriptional regulator (LTTR) that exhibits unique characteristics, suggesting that it functions as a dimer rather than the typical tetramer. Size-exclusion chromatography revealed that CutR forms a stable dimer. Electrophoretic mobility shift assays demonstrated that dimeric CutR specifically binds to an inverted repeat sequence (IR1) containing T-n12-A motifs located upstream of the *cutB* gene, which encodes the medium subunit of CO-DH. Crystal structure determination at 1.8 Å resolution revealed that CutR consists of an N-terminal DNA-binding domain with a winged helix-turn-helix motif and a C-terminal ligand-binding domain comprising two regulatory subdomains (RD1 and RD2), forming a unique two-fold symmetrical homodimer. This dimer is stabilized through four interfaces, including an extensive 12-stranded antiparallel β-sheet formed between RD1 subdomains via intertwining C-terminal β11 strands. This represents the first symmetric dimeric LTTR structure with tightly associated ligand-binding domains. The recognition helices are spaced closer together than they are in typical DNA-bound LTTRs, despite binding longer T-n12-A sequences, suggesting that a conformational change is required to enhance DNA-binding affinity. A putative ligand-binding site was identified between the RD1 and RD2 subdomains, where glycerol binding induced local conformational changes. Comparative genomic analysis revealed conservation of CutR and the IR1 sequence across *Mycobacterium* species, supporting the dimeric regulatory mechanism and providing new insights into LTTR diversity.

## 1. Introduction

**Figure 1:**
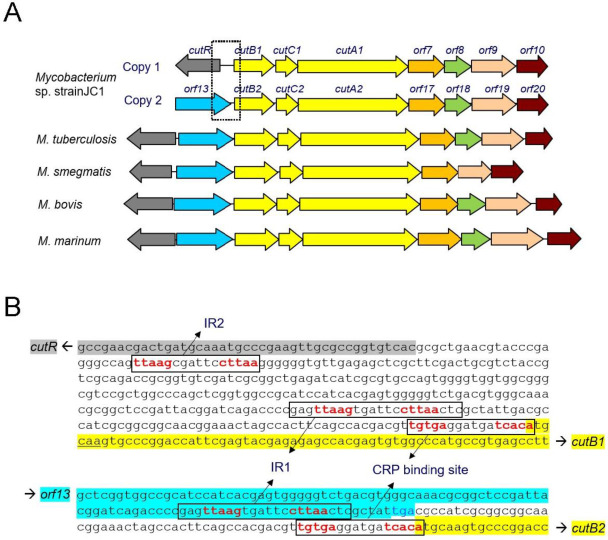
Gene architecture of CO-DH and the DNA sequences upstream of *cutB* in *Mycobacterium* sp. strain JC1. (**A**) Two copies of the genes encoding the three CO-DH subunits (yellow), along with genes involved in the biosynthesis of CO-DH cofactors, are present in *Mycobacterium* sp. strain JC1. In other *Mycobacterium* species containing a single copy of the CO-DH genes, the gene corresponding to CutR (gray) is located upstream of the gene corresponding to *orf13* (cyan). The nucleotide sequences boxed with dotted lines are shown in (**B**). (**B**) An inverted repeat sequence (IR2) is located upstream of *cutR*, while two inverted repeat sequences (IR1 and the CRP binding site) are located upstream of *cutB1* (top). The IR1 sequence upstream of *cutB1* is located at the end of *orf13* (bottom). Inverted repeat sequences are boxed, and the consensus sequences are highlighted in red. The sequence regions for *orf13*, *cutR*, and *cutB* are colored as in (**A**).

Mycobacteria species are acid-fast bacteria that can cause infectious diseases in humans [1,2]. Some pathogens, such as *Mycobacterium tuberculosis*, survive within macrophages by entering a dormant state [3,4]. In response, macrophages produce heme oxygenase, which generates toxic carbon monoxide (CO) as a defense mechanism against pathogens [5]. However, *M. tuberculosis* possesses CO dehydrogenase (CO-DH), an enzyme that oxidizes CO to derive energy [6]. In addition, CO-DH can eliminate nitric oxide (NO), another toxic molecule produced by macrophages [7]. CO-DH consists of three kinds of subunits and three cofactors: molybdopterin cytosine dinucleotide, flavin adenine dinucleotide, and an iron–sulfur center [8]. The genes encoding CO-DH including *cutB*, which codes for the medium subunit, are organized in an operon [9,10] (Figure 1A). The CO-DH operon has been extensively studied in *Mycobacterium* sp. strain JC1, and its expression is regulated by CutR, a transcriptional regulator [11]. Sequence analysis shows that CutR belongs to the LysR-type transcriptional regulator (LTTR) family. CutR-mediated transcriptional regulation is essential for the efficient utilization of CO by *Mycobacterium* sp. strain JC1.

LTTRs are composed of an N-terminal DNA-binding domain (DBD) containing a winged helix-turn-helix (wHTH) motif and a C-terminal ligand-binding domain (LBD) [12,13]. The LBD functions as a regulatory domain, composing two regulatory subdomains (RD1 and RD2); ligand binding between these subdomains induces a conformational change in the LTTRs. In the classical model of LTTR-dependent transcription regulation, the tetrameric form of an LTTR binds to both the recognition binding site (RBS) and the activation binding site (ABS) upstream of target genes in the absence of a co-inducer. Interaction with a co-inducer alters the quaternary structure of the LTTR tetramer, causing a shift of the ABS binding from site-1 to site-2—a mechanism known as “sliding dimer mechanism”—which facilitates RNA polymerase recruitment to the promoter [14,15].

CutR functions as an activator, as the expression of CO-DH increases in the presence of CutR, whereas a mutant lacking CutR exhibits a loss of CO-DH activity in *Mycobacterium* sp. strain JC1 [11]. Since glucose represses CO-DH expression, this expression is subject to catabolite repression mediated by the catabolite repression protein (CRP). However, CO-DH in *Mycobacterium* sp. strain JC1 is expressed at significant levels under non-catabolite repression conditions, regardless of the presence of CO. No dramatic increase in expression is observed upon CO exposure, suggesting that CO or its derivative is not an essential co-inducer and that CutR may function as a simple transcriptional activator without requiring a tetrameric conformational change.

*Mycobacterium* sp. strain JC1 contains duplicated CO-DH gene clusters [10], each with an active promoter located upstream of their respective *cutB* genes [11]. Within the promoter region of *cutB*, two inverted repeat (IR) sequences have been identified (Figure 1B). One corresponds to the conserved CRP binding site, while the other, designated IR1, is essential for CO-DH gene expression. IR1, situated at the −60 position, contains a conserved RBS-like sequence featuring a T-n12-A motif instead of the typical T-n11-A. However, no suitable sequence for the ABS has been identified. Therefore, CutR may function as a dimer. It has been reported that some LTTRs, such as MetR [16], CatR [17], and OccR [18], function as dimers, although the structural model for dimeric LTTRs has not yet been elucidated.

Although CutR is a member of the LTTR family, it exhibits characteristics distinct from those of typical LTTRs. The absence of IR sites that fulfill both RBS and ABS criteria upstream of *cutB* suggests that CutR does not function as a homotetramer. Furthermore, CutR can activate CO-DH gene transcription in the absence of CO, although CO enhances this expression. To elucidate the structural basis of the functional dimeric form of CutR, this study demonstrates that CutR forms a homodimer that interacts with the IR1 sequence in the absence of CO, instead of tetramer. We also present the crystal structure of CutR, revealing a dimeric LTTR with two LBDs tightly associated in a symmetrical arrangement, along with the axis of its dimeric DBD complex. This is the first structural evidence of a CutR dimer through its LBD. The structure further reveals a putative ligand-binding site capable of inducing a local conformational change.

## 2. Results

### 2.1. CutR Forms a Stable Dimer That Interacts with the Inverted Repeat Sequences Upstream of CutB

**Figure 2:**
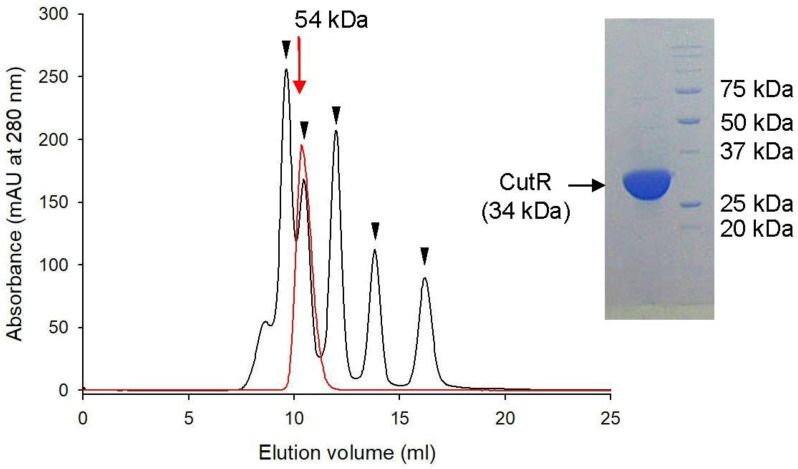
Size estimation by size-exclusion chromatography. CutR (red line) was eluted at a size corresponding to 54 kDa from a Superdex G75 column. Five proteins used as size standards (black line) are marked with inverted triangles: conalbumin (75 kDa), ovalbumin (43 kDa), carbonic anhydrase (29 kDa), ribonuclease A (13.7 kDa), and aprotinin (6.5 kDa). CutR (34 kDa) is shown as a monomeric form in the SDS-PAGE (right panel).

Since most LTTRs form tetramers through their LBDs and undergo conformational changes upon activation, the oligomeric state of CutR was first determined to establish whether it forms a dimer or a tetramer. Size-exclusion chromatography revealed that purified CutR, with a theoretical molecular weight of 34 kDa, eluted at an apparent molecular weight of 54 kDa, indicating a dimeric conformation (Figure 2). The observed molecular weight, which is lower than the theoretical 68 kDa expected for a simple dimer, suggests that CutR adopts a compact dimeric structure, distinct from the expanded conformations observed in the crystal structures of other LTTRs.

**Figure 3:**
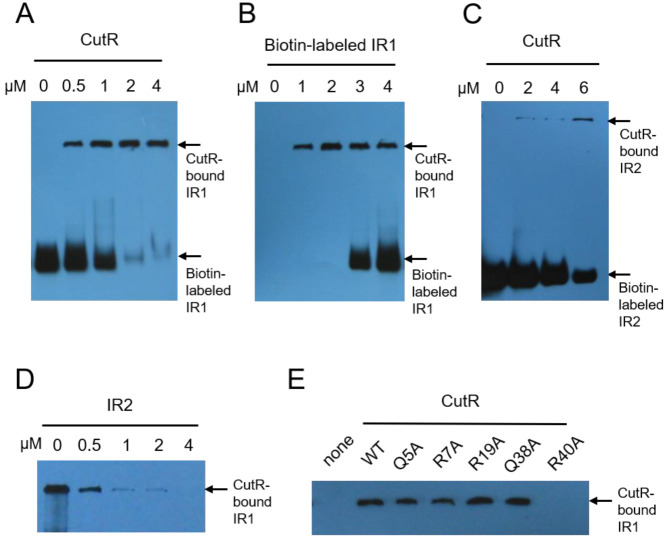
DNA-binding ability of CutR. In EMSA, CutR shifts the biotin-labeled DNA fragment containing IR1 or IR2. As the amount of CutR increases (0 to 4 μmole), the unbound DNA fragment containing IR1 at the bottom disappears (**A**), whereas increasing the DNA fragment (0 to 4 μmole) results in more unbound DNA at the bottom (**B**). (**C**) CutR shifts the biotin-labeled DNA fragment containing IR2 in EMSA. (**D**) Increasing the amount of unlabeled IR2 DNA fragments (0 to 4 μmole) causes the CutR-bound DNA fragment containing IR1 to disappear. (**E**) The CutR R40A mutant fails to bind the DNA fragment, whereas the Q5A, R7A, R19A, and Q38A mutants bind the DNA similarly to wild-type CutR.

CO-DH was expressed in *Mycobacterium* sp. strain JC1 in the absence of CO, provided that CutR was present. Therefore, the DNA-binding capability of the dimeric form of CutR was evaluated using an electrophoretic mobility shift assay (EMSA) without CO. IR1, located near the −35 region upstream of the *cutB* gene, was selected for analysis. Reporter gene assays demonstrated that the activity of the *cutB* promoter depended on CutR, and deletion of IR1 abolished reporter expression even in the presence of CutR [11]. In EMSA, dimeric CutR bound to a DNA fragment containing IR1, as evidenced by a gel shift in biotin-labeled fragments in the presence of CutR (Figure 3A,B).

Due to the duplication of the CO-DH gene cluster, *Mycobacterium* sp. strain JC1 harbors identical promoter sequences upstream of the *cutB1* and *cutB2* genes. However, the genomic contexts of these genes differ: the *cutR* gene is located divergently upstream of *cutB1*, whereas *cutB2* is adjacent to *orf13*, a gene encoding a hypothetical protein (Figure 1A). Another inverted repeat sequence (IR2), which is closely related in sequence to IR1, is situated upstream of the *cutR* gene (Figure 1B). Both IR1 and IR2 contain the TTAAG-n6-CTTAA motif. IR2 is separated from IR1 by approximately 180 bp in the *cutB1* upstream region; no IR2 was identified near *cutB2*. EMSA demonstrated that CutR also binds specifically to IR2, which features a T-n12-A motif, and that IR2 effectively competes with IR1 for CutR binding (Figure 3C,D).

To identify residues critical for DNA binding, alanine substitution mutants targeting arginine and glutamine residues within the wHTH domain of CutR were constructed. EMSA revealed that the R40A mutant was deficient in IR1 binding, whereas the Q5A, R7A, R19A, and Q38A mutants exhibited DNA binding comparable to that of wild-type protein (Figure 3E). These findings indicate that R40 is essential for IR1 recognition by CutR.

### 2.2. Crystal Structure of CutR Reveals a Two-Fold Symmetrical Dimer

**Figure 4:**
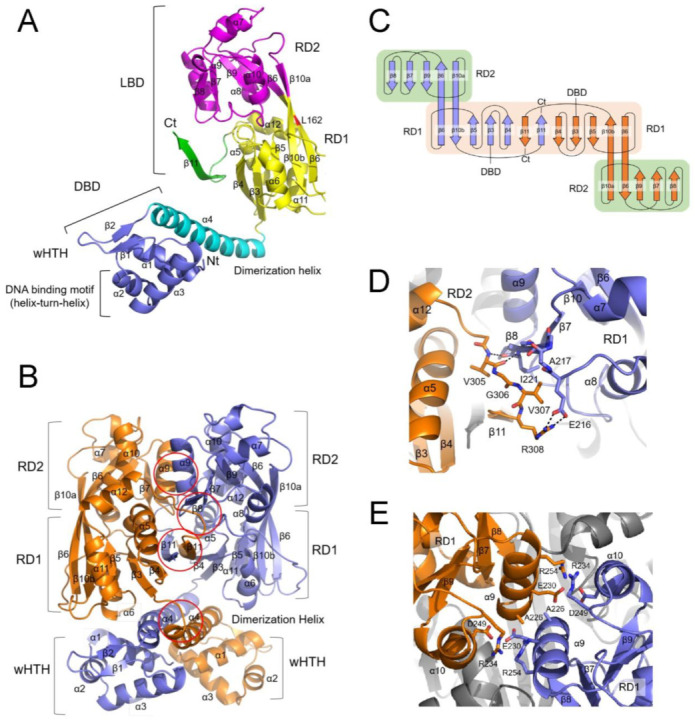
Crystal structure of CutR. (**A**) Ribbon diagram of a CutR monomer, comprising the N-terminal DNA-binding domain (DBD), which includes a winged helix-turn-helix motif (wHTH) (slate) and a dimerization helix (cyan), and the C-terminal ligand-binding domain (LBD) containing two regulatory subdomains: RD1 (yellow), RD2 (magenta), and an additional β-strand (green). The position of L162 is highlighted in red. (**B**) Ribbon diagram of the CutR dimer (slate/orange) highlighting four interacting regions (red circles), including an antiparallel interaction of the α4 dimerization helices, intertwining of the β11 strands connecting the two RD1 β-sheets, and a parallel interaction of the α9 helices in RD2. (**C**) Topology diagram of the CutR LBD dimer. Two five-stranded RD1 β-sheets form a 12-stranded β-sheet together with two β11 strands arranged antiparallel. The five-stranded RD2 β-sheet is connected to RD1 through the β6 and β10 strands. (**D**) Interaction between one RD1 and the RD2 of the other monomer is stabilized by hydrogen bonds between V305 and I221 and an ionic interaction between R308 and E216, enhancing the dimerization of the CutR LBD. (**E**) Parallel arrangement of α9 and α10 helices from both RD2 subdomains. Subdomains participating in the interaction from the two subunits are colored slate and orange. The residues at the interface are shown as sticks.

Unlike most LTTRs, CutR appears to be a compact dimer and recognizes a longer T-n12-A motif compared to the typical T-n11-A motif. To investigate the structural uniqueness of CutR, we determined its crystal structure at a resolution of 1.8 Å. Two CutR molecules were present in the asymmetric unit of the crystal, forming a stable dimer. Each monomer comprised an N-terminal DBD with a wHTH motif, followed by a long dimerization helix, and a C-terminal LBD consisting of two similar α/β subdomains designated RD1 and RD2 (Figure 4A). The RD1 subdomain contained a five-stranded mixed β-sheet (β6–β10–β5–β3–β4) plus a C-terminal strand (β11), while RD2 consisted of another five-stranded mixed β-sheet (β10–β6–β9–β7–β8). The long β6 and β10 strands connected RD1 and RD2, forming a rigid structural pillar. Notably, β6 contained a β-bulge caused by the insertion of L162, which induced a twist in the strand, rotating RD2 relative to RD1.

The two monomers interacted through four primary interfaces (Figure 4B). Dimerization was mediated by the α4 helices of the DBDs, consistent with observations in other LTTRs. Additionally, the LBDs were closely apposed; the C-terminal β11 strands from each monomer intertwined, connecting the β-sheets of the RD1 subdomains and forming an extensive 12-stranded β-sheet arranged in an antiparallel fashion with the order 6–10–5–3–4–11*–11–4*–3*–5*–10*–6* (* indicates the partner monomer) (Figure 4C). Further contacts occurred between the RD1 of one monomer and the RD2 of the other, mediated via the loop preceding β11, which interacts with the β8 strand and the loop following the α8 helix of the other monomer. Residues V305 and R308 established hydrogen and ionic bonds, respectively, with I221 and E216 in RD2 (Figure 4D). The α9 and α10 helices in each RD2 faced one another but did not form a typical hydrophobic four-helix bundle. Although the two A226 residues at the N-terminal regions of the α9 helices were closely positioned, charged residues such as E230 and R234 in the α9 helix and R254 and D249 in the α10 helices were present at the interfaces (Figure 4E).

### 2.3. Putative Ligand-Binding Site

**Figure 5:**
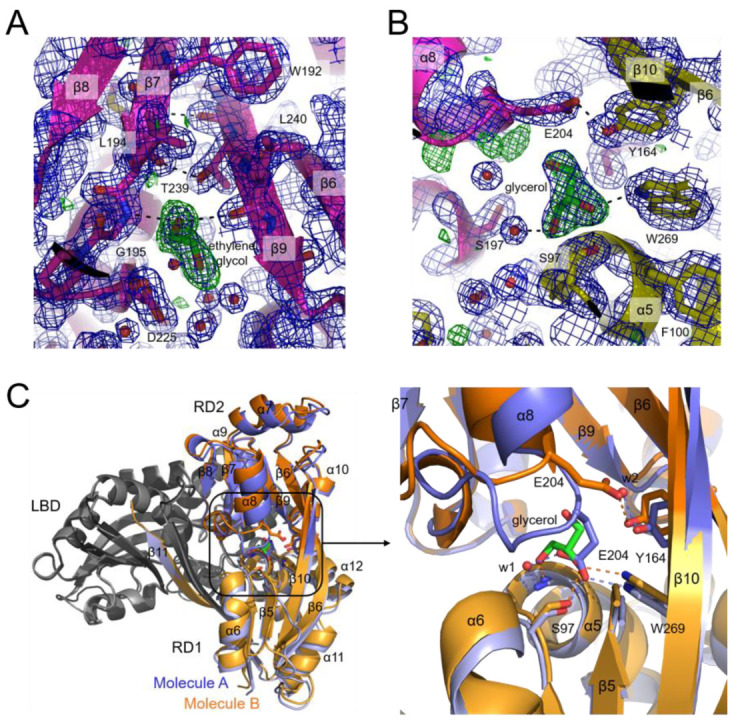
Putative ligand-binding site. (**A**) The electron density map reveals an ethylene glycol molecule (green) located between the β7 and β9 strands in the RD2 subdomain (magenta), linking their C-termini through hydrogen bonds (dashed lines). The electron density map for 2Fo-Fc at 1.5 σ contour level and the ligand-omitted Fo-Fc map at 3.0 σ contour level are colored blue and green, respectively. (**B**) A glycerol molecule (green) positioned between the RD1 (yellow) and RD2 (magenta) subdomains is surrounded by residues S97, Y164, S197, E204, and W269 in molecule B. (**C**) Two CutR LBD dimers are depicted as ribbon diagrams. Molecule A (slate) of one LBD dimer is superimposed on molecule B (orange) of the other dimer, with the other subunits in both LBD dimers colored gray. RD1 subdomains are shown in lighter shades than RD2 in both LBDs. A glycerol molecule (green) is situated between the RD1 and RD2 subdomains in molecule B. The boxed area is zoomed in the right panel. In molecule A (slate), the α8 helix and the following loop are positioned close to the α5 and α6 helices of RD2, and the side chain of E204 in the loop forms hydrogen bonds with S97 and W269. In molecule B (orange), a glycerol molecule (green) occupies the position corresponding to E204 in molecule A. The side chain of E204 in molecule B is oriented toward the β10 strand and interacts with Y164.

Within LTTRs, the ligand-binding site is typically located in the cleft between RD1 and RD2, extending into the interior of RD2. The CutR crystal structure revealed small ligands at these positions. Ethylene glycol, used as a crystallization additive, was observed sandwiched between the C-terminal ends of β7 and β8 in RD2, stabilizing the β-sheet (Figure 5A). Additionally, a glycerol molecule, used as a cryoprotectant, was detected in the cleft between the RD1 and RD2 subdomains of one monomer (molecule B) (Figure 5B). Molecule B appears more favorable for glycerol interaction due to crystal packing. To identify structural alterations, the structure of molecule B was superimposed onto that of molecule A (Figure 5C). The glycerol molecule occupies the position of the E204 side chain in molecule A. The glycerol forms hydrogen bonds with S97, G99, and W269, whereas the carboxyl group of E204 in molecule A forms hydrogen bonds with the side chains of S97 and W269, as well as with the main chain amide group of G99 via a water molecule. Due to the presence of glycerol, the carboxyl group of E204 in molecule B shifts toward the β-sheet of RD2 and interacts with the hydroxyl group of Y164, which is hydrogen-bonded to a water molecule in molecule A. This glycerol-induced rearrangement alters the local conformation of the loop containing E204 and causes subsequent movement of the α6 and α8 helices.

### 2.4. Structure of the Winged Helix-Turn-Helix Motif

**Figure 6:**
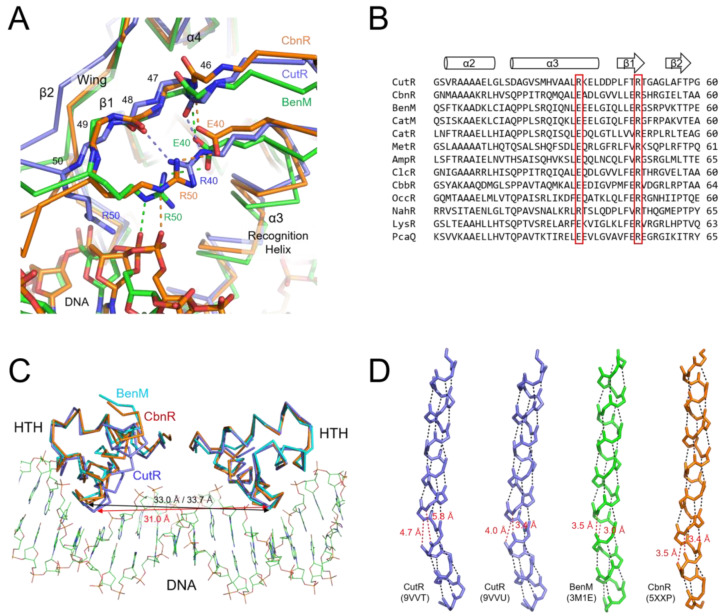
Structure of DNA-binding domains. (**A**) The position of the wing, consisting of the β1 and β2 strands, is stabilized through the interaction of R40, which contacts the main chain of the β1 strand from the α3 recognition helix in CutR (slate). In the CbnR (orange) and BenM (green) structures, E40 from the α3 helix stabilizes the β1 strand. R50 from the β1 strand interacts with the phosphate backbone of DNA. Hydrogen bonds, shown as dashed lines, are colored according to their respective proteins. (**B**) Glutamate or arginine at the α3 helix and arginine at the β1 strand are conserved in all LTTRs. (**C**) The distance between the α3 recognition helices in the CutR dimer (slate) is shorter than those in the DBD dimers of CbnR (orange) and BenM (cyan). The red and black arrows indicate the distances between two A30 residues of CutR and two P40 residues in BenM or CbnR, respectively, recognizing the thymine bases in the conserved T-n11-A DNA sequence. (**D**) The curvature of the α4 dimerization helix results from a kink in the helix. More gaps in the broken hydrogen bonds within the CutR helix increase its curvature, thereby decreasing the distance between the two recognition helices in a DBD dimer. Hydrogen bonds within the helix are shown with dashed lines; broken hydrogen bonds are highlighted in red. The PDB IDs are provided in parentheses.

The DBD of CutR comprises a canonical wHTH motif and a dimerization helix. The DNA-binding motif is formed by the α2 and α3 helices, followed by a β-hairpin that contributes to interactions with the DNA backbone. The R40 residue, located on the α3 recognition helix, is critical for DNA binding, as demonstrated by the loss of binding in the R40A mutant. Structural analysis reveals that R40 interacts with the main chain carbonyl oxygens of P46 and F48, stabilizing the orientation of the wing relative to the recognition helix and supporting productive interactions with DNA phosphates (Figure 6A). At the equivalent position to R40, a glutamic acid residue is conserved in most LTTRs (Figure 6B). In the crystal structures of BenM [19] and CbnR [20], E40 stabilizes the position of the wing through an interaction with the amide group of residues N47 (Figure 6A). Due to the difference in charge of R40 in CutR, the peptide backbone at P46 is flipped compared to that in BenM and CbnR.

The spacing between the two recognition helices of the CutR dimer was slightly shorter than that observed in the crystal structures of BenM and CbnR, which are complexed with DNA (Figure 6C). In CutR, the distance between the Cα atoms of A30—corresponding to P30 in BenM and CbnR, which interacts with thymidine in the T-n11-A motif—is 32.4 Å (31.0 Å in the selenomethionine-substituted CutR). In contrast, BenM [19] and CbnR [21] exhibit distances of 33.7 Å and 33.0 Å, respectively, despite CutR binding to a longer T-n12-A motif. This suggests that the CutR dimer binds two consecutive major grooves on the same side of B-form DNA, potentially inducing DNA bending.

An appropriate distance between two recognition helices can be achieved by extending the α4 dimerization helices, which position the wHTH motifs at their termini. The length of the dimerization helix depends not only on the number of helical turns but also on its curvature. The N-terminal region of the α4 helices is slightly bent due to disruption of hydrogen bonds within the helix, typically at the third turn (Figure 6D). Broken hydrogen bonds with distances of approximately 3.5 Å were observed in the structures of BenM and CbnR. In the CutR structure, there is a pronounced kink in the helix, with disrupted hydrogen bonds separated by about 5.0 Å. This kink appears to bring the two wHTH motifs closer together.

## 3. Discussion

**Figure 7:**
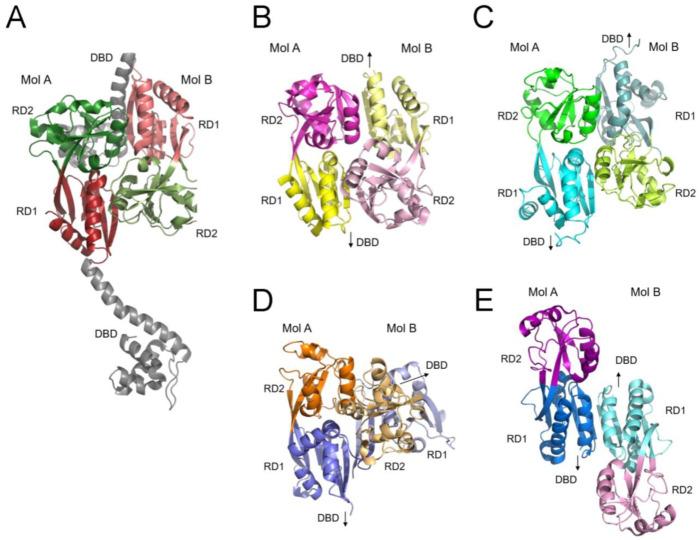
Dimeric structures of the ligand-binding domains. Arrangements of the four RD subdomains, each distinctly colored, in an LBD dimer are shown as ribbon diagrams from the tetrameric CbnR complex exhibiting separated DBDs (gray) (**A**); the crystal structures of LBD dimers of CynR (**B**) and Cbl (**C**); the dimeric CutR structure (**D**); and the crystal structure of the LBD dimer of MvfR in the absence of the C-terminal tail (**E**). Molecules B are depicted in lighter shades than molecules A in the LBD dimers. There is no interaction between the two RD1 subdomains of CynR and Cbl, whereas CutR and MvfR exhibit RD1-RD1 interactions. Twisting of the connected RD1 β-sheets facilitates contact between the two RD2 domains and brings the two DBDs into close proximity in the CutR dimer. Arrows indicate the location of the DBD.

It is well established that LTTRs bind DNA as dimers through their α4 dimerization helices, and that two dimeric LTTRs can associate via their LBDs to form tetrameric LTTR complexes. Structural studies of tetrameric LTTRs have revealed asymmetric arrangements of their LBDs within homodimeric DBD units, alongside a two-fold symmetric arrangement of LBDs from different DBD pairs as shown in the crystal structure of CbnR [22] (Figure 7A). However, to date, no dimeric structure featuring a symmetric LBD arrangement together with a two-fold symmetric DBD dimer has been reported. Symmetric dimeric arrangements of LBDs have been observed in crystal structures of isolated LBDs, such as those of CynR [23] and Cbl [24]. In these dimers, the β-sheets from RD1 and RD2 are connected in a parallel manner (Figure 7B,C). The close contact between RD1 and RD2 subdomains resembles the arrangement of RD subdomains in the DBD pairs of tetrameric LTTRs. This configuration, however, cannot support the characteristic antiparallel interactions of the dimeric DBD, which are necessary for the correct positioning of the two dimerization helices.

In this study, we present the homodimeric, full-length structure of CutR, a representative of the LysR family. The structure is characterized by a pronounced twisting of the two LBDs, stabilized by the intertwining of the C-terminal β-strand, which results in extensive interactions between the two RD1 subdomains (Figure 7D). Other LTTRs, such as MetR [16] and CbbR [25], which function as dimers, may adopt similarly stable homodimeric structures facilitated by their extended C-terminal peptide regions, as observed in CutR. LTTRs with longer sequences than typical members contain flexible C-terminal tails in their monomeric structures, as suggested by AlphaFold predictions [15]. PqsR (MvfR) also contains a long C-terminal extension [26], likely corresponding to the terminal β-strand (β11) in CutR. Notably, the crystal structure of the PqsR LBD lacking this C-terminal tail (PDB ID: 7QA0), in complex with a ligand [27], demonstrates dimer formation via antiparallel interactions between two RD1 β-sheets (Figure 7E). The presence of two additional β-strands between the RD1 sheets could facilitate greater rotation of the RD2 subdomains by twisting the β-sheet, enabling further interactions between RD2 subdomains. Thus, these LTTRs containing compact dimeric LBDs require an induction mechanism other than ligand-induced tetramerization.

Most LTTRs recognize a conserved T-n11-A sequence, although some bind to modified variants. For example, OccR [28] binds a shorter T-n10-A sequence, while PcaQ [29] and IlvY [30] recognize T-n12-A and T-n13-A sequences, respectively. The conserved thymine bases within the inverted repeat are typically recognized by a residue at position 30 in the recognition helix, as observed in the BenM [19] and CbnR [21] structures complexed with their target DNA containing the T-n11-A motif. CutR specifically binds to the IR1 and IR2 sequences containing the T-n12-A motif. In CutR, residue A30, located at the N-terminus of the α3 recognition helix, corresponds to P30 in BenM and CbnR, which recognize the thymine bases. For A30 to interact with both bases, the recognition helices would be expected to be spaced farther apart than in LTTRs that bind the canonical T-n11-A sequence. Surprisingly, structural comparison revealed that the two recognition helices in CutR are actually closer together. This suggests that increasing the distance between the recognition helices—potentially through by local conformational changes near the wHTH motif—may be necessary to optimally recognize the T-n12-A motif.

Although DNA bending and induced fit of the DBD may facilitate the accommodation of the recognition helices within the DNA, the LBD also appears to influence the positioning of the wHTH motif. The distance between the Cα atoms of residue P30 in full-length BenM without DNA (PDB ID: 3K1N) is 31.4 Å, increasing to 33.1~33.7 Å in the presence of DNA (PDB IDs: 4IHS and 4IHT). The LBD affects the position of the wHTH motif, as the structure of the DBD alone (PDB ID: 3M1E) shows a distance of 33.0 Å. The full-length CbnR complexed with DNA displays a distance of 33.4 Å (PDB ID: 7D98), whereas the DBD-DNA complex of CbnR (PDB ID: 5XXP) has a distance of 32.7 Å.

The DNA-binding affinity of CutR is determined by the positioning of the wHTH motifs, including the α3 recognition helices. Both the length and curvature of the α4 dimerization helix influence the spacing between the two wHTH motifs. Since the LBD pair in the CutR dimer is connected to the wHTH motifs through the α4 helix, changes in the arrangement within the LBD pair can affect the bending of the α4 dimerization helices, thereby modulating DNA binding. The crystal structure reveals that glycerol interaction within the cleft between the RD1 and RD2 subdomains can alter the local conformation of these subdomains, including the α6 helix adjacent to the wHTH motif. Optimal positioning of the α3 recognition helices induced by ligand binding could enhance CutR’s affinity for its target DNA.

CutR functions as a constitutive activator of CO-DH expression by forming a compact dimer capable of binding DNA even in the absence of a ligand. Although the distance between its recognition helices is shorter than that observed in DNA-bound LTTRs, CutR still binds its target DNA efficiently. Consistent with this, CO-DH is expressed at a significant baseline level in *Mycobacterium* sp. strain JC1 regardless of CO presence, provided that CutR is present. However, CO exposure further enhances CO-DH expression, indicating that ligand binding may induce subtle structural changes in CutR that increase its affinity for the IR1 site, thereby boosting transcriptional activation. Although CO is usually recognized directly by heme-based regulators such as CooA [31] and RcoM [32], CutR appears to achieve this through the binding of a small molecule at the site of residue E204. Since *Mycobacterium* sp. strain JC1 can utilize various C_1_ compounds [6], CutR may recognize a metabolite generated in the presence of CO. However, we cannot rule out the involvement of another regulatory protein in recognizing CO.

Genomic sequence analysis of *Mycobacterium* sp. strain JC1 reveals two copies of the CO-DH gene, resulting from a gene cluster duplication. Both *cutB* genes (*cutB1* and *cutB2*) contain the CutR target sequence IR1 in their upstream regions. CO-DH expression is subject to catabolic repression, as indicated by the presence of a CRP binding site near the promoter regions of both *cutB* genes, adjacent to IR1. Additionally, IR2 is located upstream of *cutB1*, likely due to the proximity of the *cutR* gene. Functional analyses indicate that while both *cutB1* and *cutB2* promoters are active, *cutB2* is more strongly activated by CutR, suggesting that IR1 alone is sufficient for CutR-mediated activation of the *cutB* genes.

Comparative genomic analysis reveals that most *Mycobacterium* species possess a CO-DH gene cluster analogous to the *cutB2* locus in *Mycobacterium* sp. strain JC1 (see Figure 1A). The *cutR* gene is highly conserved, particularly in its wHTH motif and key residues of the putative ligand-binding site, including S97, Y164, E204, and W269. All examined species retain a conserved IR motif containing a T-n12-A sequence, designated IR1, which serves as the CutR binding site, although upstream promoter regions may vary. Some species (e.g., *M. tuberculosis*, *M. bovis*, *M. marinum*) lack the CRP binding motif, whereas others (e.g., *M. smegmatis*) share this feature with *Mycobacterium* sp. strain JC1 [11]. These findings support the hypothesis that CutR functions as a dimer, interacting with the IR sequence to regulate and activate the CO-DH gene cluster across *Mycobacterium* species.

## 4. Materials and Methods

### 4.1. Cloning, Expression, and Purification

The gene encoding CutR from *Mycobacterium* sp. strain JC1 was amplified by polymerase chain reaction (PCR) from the pCUTR plasmid [11] using a forward primer (5′-GATCCATATGACACCGGCGCAACTT-3′) containing an *Nde*I site and a reverse primer (5′-TCAGGGATCCTCAGTGGTGGTGATGGTGGTGGCTCCAGAGCGTGACGTGG-3′) that includes six His codons before the stop codon and a *Bam*HI site. The amplified fragment was digested with *Nde*I and *Bam*HI and ligated into the expression vector pT7-7. Site-directed mutagenesis was performed to generate the Q5A, R7A, R19A, Q38A, and R40A mutants using the QuikChange method, and all mutations were confirmed by sequencing.

To obtain the CutR protein, the recombinant plasmid pT7-7: *cutR* was co-transformed with the pGro7 chaperone plasmid, which carries the GroES and GroEL genes, into *Escherichia coli* strain BL21 (DE3). The transformant cells were grown at 37 °C in LB medium, and expression of the CutR protein was induced at the logarithmic phase (OD_600_ = 0.5) by adding 0.2 mM isopropyl β-d-thiogalactoside. The cells were then cultured for an additional 20 h at 18 °C. Harvested cells were resuspended in Buffer A (50 mM Tris–HCl, pH 7.5, 500 mM NaCl, and 5 mM β-mercaptoethanol) and disrupted by sonication. Cell debris was removed by centrifugation and the soluble supernatant was applied to a Ni-NTA agarose (QIAGEN) column. After washing with Buffer A containing 30 mM imidazole, the bound proteins were eluted with 200 mM imidazole in buffer A. Purified CutR protein was subjected on size exclusion chromatography using a Superdex G-75 column pre-equilibrated with 20 mM Tris-HCl, pH 7.5, 500 mM NaCl, and 5 mM β-mercaptoethanol. The selenomethionine (SeMet)-substituted CutR protein was expressed in the methionine auxotroph *E. coli* B834 (DE3) strain in M9 minimal medium supplemented with 50 mg/mL SeMet at 25 °C. The purification of the SeMet-substituted protein was identical to that of the native protein.

### 4.2. Crystallization and Data Collection

The recombinant CutR protein was concentrated to 26 mg/mL and subjected to initial crystallization screening using the hanging-drop vapor diffusion method at 21 °C with commercially available sparse-matrix screens from Hampton Research and Emerald Biostructures. After optimization via microseeding, the best crystals were obtained using 5% (*w*/*v*) PEG 8000, 0.1 M sodium acetate (pH 4.6), and 4% (*v*/*v*) ethylene glycol. Crystals were transferred to reservoir solution containing 20% glycerol as a cryoprotectant prior to X-ray data collection. Following a fluorescence scan, single anomalous X-ray dispersion data for a SeMet-labeled crystal were collected at the selenium absorption peak wavelength (0.97926 Å) using an ADSC Quantum 210 CCD area detector on beamline 4A at the Pohang Accelerator Laboratory (PAL, Pohang, Republic of Korea). Data for native CutR were collected at a wavelength of 0.97933 Å on the PAL 7A beamline. The data were indexed, integrated, and scaled using the HKL2000 package [33]. Both SeMet and native crystals belong to the space group *P2_1_2_1_2_1_* and their unit cell parameters indicate two CutR molecules in the asymmetric unit. Crystal statistics are summarized in Table 1.

### 4.3. Structure Determination

The structure of SeMet-substituted CutR was determined by exploiting the anomalous signals from selenium atoms using the program SOLVE [34], which identified twelve Se sites in the asymmetric unit. The overall figure of merit (FOM) was 0.357. Density modification and subsequent automated model building were performed using the program RESOLVE [35], which increased the FOM to 0.647 and built 74% (487 of 654) of the amino acid residues. Further model building was conducted manually using the program COOT [36], and refinement with isotropic displacement parameters was carried out using REFMAC5 within the CCP4 suite [37]. Atomic displacement parameters were refined using the TLS method [38], with each of the two monomers in the asymmetric unit treated as a single TLS group. The R_work_ and R_free_ values of the refined structure were 0.212 and 0.268, respectively. The structure of native CutR was solved by molecular replacement using Phaser [39], employing the previously determined SeMet-substituted CutR structure as a template. COOT and PHENIX [40] were used for model building and refinement. Crystallographic data statistics are summarized in Table 1. The atomic coordinates and structure factor amplitudes of the SeMet-substituted and native proteins were deposited in the Protein Data Bank under accession codes 9VVT and 9VVU, respectively.

### 4.4. Electrophoretic Mobility Shift Assay (EMSA)

EMSA experiments were performed using the Chemiluminescent Nucleic Acid Detection Module Kit (Thermo Scientific, Waltham MA, USA). Target DNA fragments were derived from the IR1 to CRP motif (101 bp) and IR2 (29 bp) regions of the upstream promoter of the *Mycobacterium* sp. strain JC1 CO-DH gene. The IR1 region was amplified from *Mycobacterium* sp. JC1 genomic DNA by PCR using a forward primer (5′-biotin-ACCCCGAGTTAAGTGATTCC-3′) and a reverse primer (5′-TTGCATGTGATCATCCTCAC-3′). The IR2 DNA fragment was generated by annealing equimolar amounts of complementary synthetic oligonucleotides, including a biotin-labeled forward strand (5′-biotin-GGGCCAGTTAAGCGATTCCTTAAGGGGGG-3′) and its complementary reverse strand (5′-CCCCCCTTAAGGAATCGCTTAACTGGCCC-3′). PCR products and annealed oligonucleotides were purified by gel extraction. For binding reactions, 0.8–3.2 μmol of biotin-labeled target DNA was incubated with 1.425–11.4 μmol of purified CutR protein in 10 μL of binding buffer (50 mM Tris-HCl, pH 8.0, 100 mM NaCl, 40% glycerol, 5 mM DTT, 2 mM EDTA). The reactions were incubated at 4 °C for 1 h. The mixtures were then loaded onto a 10% native polyacrylamide gel prepared in 0.5× TBE buffer and electrophoresed at 80 V for 170 min at 4 °C. Subsequently, biotin labeling was detected by Western blotting.

## Figures and Tables

**Table 1:** Data collection and refinement statistics.

Data Set	SeMet	Native
Experimental Data		
X-Ray source	PAL 4A	PAL 7A
Wavelength (Å)	0.97926	0.97933
Space group	*P2_1_2_1_2_1_*	*P2_1_2_1_2_1_*
Unit cell parameters		
*a*, *b*, *c* (Å)	63.50, 92.08, 126.19	69.93, 81.81, 101.06
α, β, γ (°)	90.00, 90.00, 90.00	90.00, 90.00, 90.00
Resolution limit (Å) Total reflections Unique reflections Redundancy Completeness (%)	50–2.50 (2.59–2.50) ^a^ 378,265 26,311 14.4 (14.6) 100 (100)	50–1.80 (1.83–1.80) 337,472 52,582 6.4 (3.8) 96.5 (76.4)
R_symm_^b^	0.117 (0.492)	0.062 (0.569)
Average I/σ (I) CC_1/2_	43.4 (6.2) -	37.8 (3.0) 0.999 (0.927)
Refinement Details		
Resolutions (Å)	40.26–2.50	36.62–1.80
Reflections (working) Reflections (test)	23,187 1252	50,949 1949
R_work_ /R_free_ ^c^	0.212/0.268	0.191/0.233
Number of atoms Proteins Ligands Waters	4889 0 44	4797 14 398
RMSD		
Bond length (Å)	0.008	0.005
Bond angle (^o^) Average B factors (Å^2^)	1.115	0.736
Mol. A (main/side) Mol. B (main/side) Water molecules Ramachandran plot Favoured (%) Allowed (%) Outliers (%)	35.95 (35.73/36.21) 39.08 (39.02/39.14) 34.30 96.0 3.7 0.3	33.06 (30.22/36.31) 34.19 (37.05/31.72) 32.37 98.1 1.9 0.0

^a^ The numbers in parentheses describe the relevant value for the highest resolution shell. ^b^ R_sym_ = Σ|I_i_-<I>|/ΣI where I_i_ is the intensity of the i-th observation and <I> is the mean intensity of the reflections. ^c^ R_work_ = Σ||F_obs_|−|F_calc_||/Σ|F_obs_|, crystallographic R factor, and R_free_ = Σ||F_obs_|-|F_calc_||/Σ|F_obs_| when all reflections belong to a test set of randomly selected data.

## Data Availability

The original data are available from the corresponding author upon request.
